# Research on Performance Deterioration of Internally Cured Pavement Concrete under the Coupling Effect of Salt Freeze–Thaw

**DOI:** 10.3390/polym15030476

**Published:** 2023-01-17

**Authors:** Jieting Xu, Xiao Qin, Yongkang Lin, Chaofeng Cao, Junhong Liu, Qingjian Huang

**Affiliations:** 1 Advanced and Sustainable Infrastructure Materials Group, School of Transportation and Civil Engineering and Architecture, Foshan University, Foshan 528225, China; 2 The Building Construction Co., Ltd. of CTCE Group, Hefei 230601, China

**Keywords:** internally cured pavement concrete, super absorbent polymer (SAP), performance deterioration, salt freeze–thaw resistance, pore structure characteristics, hydration characteristics

## Abstract

This paper aims at solving the material durability problem caused by spraying deicing salt on pavement concrete in the northern winter. Super absorbent polymer (SAP) was adopted as an internal curing agent to enhance the durability of pavement concrete. Curing parameters including particle size and dosage of SAP and curing condition were optimized based on mortar tests by means of the grey target decision method. The deterioration rule of durability and mechanical properties of pavement concrete internally cured by different SAP dosages after salt freeze–thaw cycles were explored through rapid freeze–thaw test. Combined with the characteristics of pore structure, hydration and microstructure, the influence mechanism of SAP on the salt freeze–thaw resistance of pavement concrete was revealed. The experimental results showed that: (i) The reduction in mass loss rate and relative dynamic modulus was significantly improved by SAP internal curing with moderate dosage; (ii) The more freeze–thaw cycles the specimen underwent, the greater the increase in strength; (iii) After 75 cycles, the chloride ion erosion depth could be decreased by approximately 23.18%. Moreover, the addition of SAP could refine the pore size, inhibit the generation of shrinkage microcracks, and promote the degree of cement hydration in the late stage, which improved the internal density of the cement concrete structure. Therefore, the deterioration of pavement under the coupling effect of salt freeze–thaw was reduced.

## 1. Introduction

In order to improve the safety of driving on icy roads in the northern winter, de-icing salt (sodium chloride) is usually sprayed to melt the snow and ice. However, the values of surface chloride deposition may increase in winter; meanwhile, salt solution may penetrate into the internal structure of pavement concrete through cracks and pores, which probably causes the salt-freeze erosion disease [1].

In fact, more than 80% of the structural cracks in pavement are contributed by non-loaded cracks. Non-loaded cracks are generated frequently due to the water consumption during the hydration process and the water evaporation of concrete in the early stage of curing, which provide several channels for chlorine ions to enter the internal structure. In the meantime, the durability of pavement is probably affected by the pore structure characteristics of concrete. Therefore, an effective method is urgently needed to inhibit microcracks and optimize the pore structure of concrete to enhance the salt freeze–thaw resistance of pavement concrete.

Internal curing technology can reduce cracks due to shrinkage and improve the durability of cement-based materials [2,3]. SAP is an effective internal curing agent, which can store water in advance and release water in time when the ion concentration and humidity inside concrete decrease during the curing period. Accordingly, early autogenous shrinkage and drying shrinkage cracks are restrained effectively. In addition, SAP possesses the advantages of no pollution and low cost, which conforms to the principle of sustainable development, so that the social, economic and environmental issues are better balanced. The above advantages of SAP internal curing lay a good foundation for its promotion in practical engineering [4,5,6,7,8].

Most studies have shown that SAP internal curing can improve the mechanical and impermeability properties of cement-based materials, and there is an optimal dosage of SAP. It indicates the feasibility of studying the durability of SAP-concrete in experiencing environmental effects [9,10,11]. Regarding the above conditions, the investigation of salt freeze–thaw resistance of internally cured pavement concrete has application significance for practical engineering. Nevertheless, considerable attention has been paid to the water freeze resistance in most of the existing research, but few concentrate on salt freeze–thaw relatively. The specific status of studies on this theme is described as follows: Previous research has shown that the freeze resistance of cement-based materials internally cured by SAP could be improved [6]. In the research of Mechtcherine [12], a much less significant decrease in the dynamic Young’s modulus was found in freeze–thaw testing without de-icing salt, benefiting from the addition of SAP. Meanwhile, the effect of freeze resistance was closely related to SAP particle size. The literature reported that with the same dosage, the SAP-concrete with particle size less than 63μm developed approximately twice as much scaling as the concrete with SAP in the size range from 63 to 125 μm after 28 freeze–thaw cycles [13].

The above studies indicated that the addition of SAP could bring a positive effect on the freeze–thaw resistance of concrete, which is attributed to the optimization of pore structure. The effect of internal curing by SAP on pore structure mainly contains the following two aspects. The first was the appropriate increase of porosity. Related studies demonstrated that a quantity of closed spherical pores inside the structure would be produced by SAP gels that had released water. In this case, the air content of concrete was increased, which could protect concrete from freezing damage [14,15,16,17]. In contrast, the strength of concrete might be negatively affected owing to excessive pores generated by SAP particles [18]. The second was the refinement of pore structure. Dang et al. [19] found that the gel pores and small capillary pores were increased while the big capillary pores and air pores were reduced during the internal curing process. Particularly, the number of pores smaller than 300 μm could be increased [20]. Furthermore, Ma [21] attributed the decrease of pore size to the formation of a hydration diffusion layer, and the larger the size of SAP particles, the better the effect at day 28. It is worth mentioning that the additional internal curing water cannot be ignored, and was considered as an important factor affecting the micropore structure of concrete [22].

Moreover, it is meaningful to explore the impact of SAP internal curing on the hydration characteristics of cementitious materials. Li et al. [23] researched the hydration rate of a specimen internally cured by SAP with the dosage of 0.5%, and found that the hydration rate of the control group was approximately equal to or greater than internal curing group before 13.5 h, but it was overtaken by the latter after 13.5 h. Justs et al. and Liu et al. [24,25] reported that SAP internal curing delayed the main hydration peak, and the cumulative heat after 15 h was significantly increased, thus the degree of hydration was increased. Additionally, the related result showed that after the curing age (28 days), the area of the Ca(OH)_2_ absorption peak in the Fourier transform infrared spectroscopy (FTIR) spectrum of SAP-concrete was 3.38 times higher than that of the control group [26]. The amount of hydration product was increased to fill the residual pores formed by SAP gels, which could reduce the strength loss. Furthermore, the internal curing effect could promote the hydration process of unhydrated minerals to produce a calcium silicate hydrate (C-S-H) gel, which enhanced the compactness of the material [27,28,29]. Qin et al. [30] found that the residual pores formed by the smaller-sized SAP gels would be filled well by hydration products and the bonding strength of the interface transition zone (ITZ) between the aggregate and cement paste was enhanced.

In summary, SAP internal curing is beneficial to improve the characteristics of pore structure and hydration of cement-based materials, which may help to mitigate the damage of concrete under the coupling effect of salt and freeze–thaw. Meanwhile, previous studies have shown that the internal curing effect of SAP on concrete was highly sensitive to the amount of internal curing water that additional added, which was also affected by raw materials and the engineering environment. Consequently, optimal performance might not necessarily be achieved while choosing the theoretical amount of internal curing water, which could possibly have an influence on the exploration of the performance deterioration rule of pavement concrete under the coupling effect of salt and freeze–thaw. Therefore, it is necessary to find the optimum amount of internal curing water and dosage of SAP through reliable methods. At the same time, to evaluate the results more objectively, mathematical methods are applied, which also can be seen in the existing literature [31]. Otherwise, little attention has been paid to the pore structure characteristics and much less on the hydration characteristics of cement-based materials after salt freeze–thaw cycles. Both of them play an important role in the study of the influence mechanism of SAP internal curing on salt freeze–thaw resistance. Hence, the microstructure of internally cured pavement concrete after environment cycles should be further investigated. Furthermore, the method of analyzing test results of FTIR and SEM mostly adopted qualitative analysis, which made the results not intuitive enough.

In this work, curing parameters were optimized based on the grey target decision method via the mortar tests. The internal curing effect of SAP is greatly affected by its amount. When the dosage is insufficient, the curing effect is not obvious, but when the dosage is too much, it will produce more pores. To get the SAP optimal dosage more accurately, the performance of SAP-concrete was studied by reducing the dosage range of SAP after optimization. A rapid freeze–thaw test adopting NaCl solution was carried out to simulate the salt freeze–thaw coupling environment the pavement concrete undergoes. The performance deterioration of SAP-concrete with different SAP dosages under the coupling effect was evaluated by the indicators of mass loss rate, relative dynamic elastic modulus, flexural strength, compressive strength and chloride ion erosion depth. In particularly, the pore structure characteristics of concrete were studied by mercury intrusion porosimetry (MIP). In addition, isothermal titration calorimetry (ITC) and FTIR were conducted to research the hydration characteristics of internal curing concrete at different environmental erosion stages. Furthermore, scanning electron microscopy (SEM) was used to analyze the microstructure of the concrete before and after undergoing the salt freeze–thaw cycles. At the same time, the pores in the micromorphology were quantitatively analyzed by the Image J software. Based on the characteristics of pore structure, hydration and microstructure, the influence mechanism of SAP on the salt freeze–thaw resistance of pavement concrete was revealed.

## 2. Materials and Mix Proportion of Mortar

### 2.1. Super Absorbent Polymer (SAP)

**Figure 1:**
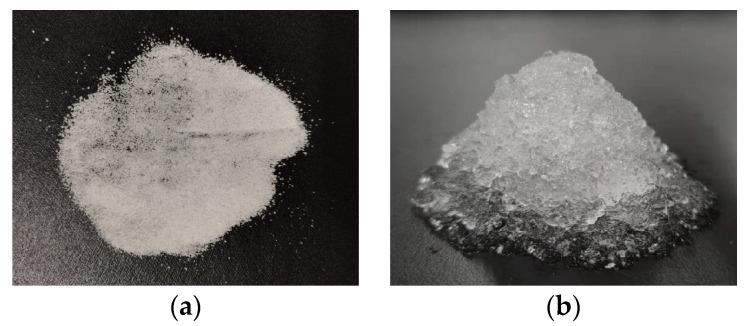
Macroscopic appearance of SAPs: (**a**) Dry SAPs; (**b**) SAP gels after water absorption.

**Figure 2:**
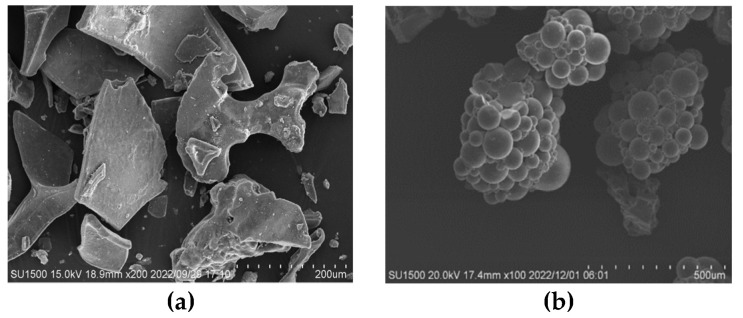
Morphology of SAPs: (**a**) Dry SAPs; (**b**) SAP gels after water absorption.

Polyacrylate-sodium SAP from Shandong Huadi Co., Ltd. in Shandong Province of China was used as internal curing agent in this study, which included two particle sizes of 100–120 mesh (SAP-100) and 150–200 mesh (SAP-200). The macroscopic appearance and morphology of SAPs before and after absorbing water are presented in Figure 1 and Figure 2, respectively. The main technical indicators and the measured liquid absorption in fresh cement paste with a *W*/*C* (water–cement ratio) of 0.4 at 30 min of SAP are illustrated in Table 1.

### 2.2. Cement and Aggregates

Table 2 lists the physical and mechanical properties of the ordinary Portland cement (PO.42.5) used in this research. The relative content of SiO_2_, Al_2_O_3_, Fe_2_O_3_, CaO, MgO and SO_3_ compositions of the cement was 24.99%, 8.26%, 4.03%, 51.42%, 3.71%, 2.51% by mass, respectively. To meet the requirements of gradation design and reach a better compactness, the coarse aggregate (granite) was divided into two particle sizes of 5–10 mm and 10–20 mm, which proportionally was 30% and 70%, respectively. In addition, river sand was employed as fine aggregate with the fineness modulus of 2.80.

### 2.3. Water Reducer and Water

Polycarboxylate-type high-performance water-reducing agent with the reduction rate of 26% and the recommended dosage of 0.8% to 1.2% was employed, which could improve the workability of cement-based materials. Experimental water was Foshan tap water.

### 2.4. Mix Proportion of Mortar

The theoretical amount of internal curing water (IC water) and theoretical dosage of SAP (by mass of cement) could be determined both by the SAP’s liquid absorption (as seen in Table 1) and the powers formula shown in Equation (1). In the pre-experiment, it was found that SAP with large particle size would leave relatively large residual pores after water release and it was difficult to be well filled by hydration products, which might affect the performance of cement-based materials. Therefore, in this experiment, two kinds of SAP—SAP-100 and SAP-200—with relatively small particle size were selected. As for the effect of dosage, the pre-experiment found that the curing effect of less than 1% dosage was not obvious. Based on the above, three kinds of dosage of SAP from 0.10% to 0.20% were chosen in this study. In addition, there are two methods of surface curing treatment for specimens, including with and without the water-resisting agent. The mix proportions of cement mortar are listed in Table 3. It was worth mentioning that the dosage of water-reducing agent in the mix proportion of mortar was determined according to the recommended dosage and the jump table test of cement mortar.

(1) $$\begin{array}{l} W/C\leq 0.36,\;{\left(W/C\right)}_{IC}=0.18(W/C)\\ 0.36\leq W/C\leq 0.42,\;{\left(W/C\right)}_{IC}=0.42-(W/C) \end{array}$$

where (*W*/*C*)*_IC_* is the additional water–cement ratio required for internal curing.

Where, SAP-1-0.10% means the internal curing group with SAP-100 and the dosage of SAP is 0.10% under natural curing condition, while WRA means that the curing condition of the specimen sealed with a layer of water-resisting agent.

In order to make sure that the SAP was dispersed uniformly in mortar, the mixing steps were as follows:

Step 1. Dry-mixing the fine aggregate and cement. (20 s)
Step 2. Adding SAP powder and dry-mixing the mixture. (30 s)
Step 3. Adding water, water reducer and internal curing water, then wet mixing the mixture. (90 s)

## 3. Experiment Methods

### 3.1. Mechanics and Shrinkage Test of Cement Mortar

The mortar specimens were prepared according to the mixing proportions illustrated in Table 3. After molding, the mortar specimens were placed in the environment of 80%RH and 25 ± 2 ℃. According to T0506-2005 cement mortar strength test method in JTG 3420-2020 [32], the mechanical properties of mortar were tested by flexural and compressive strength machine, respectively. Specimens with specification of 40 mm×40 mm×160 mm were prepared as research carrier for internal curing, which contained three similar specimens in each group.

The shrinkage performance of mortar was determined based on the dry shrinkage test method of cement mortar in JTG 3420-2020 [32], the size of the mortar specimen was 25 mm×25 mm×280 mm in this test. Curing conditions of specimens were the same as mechanical tests. The shrinkage rate measured on the digital display length meter was taken as the evaluation index of shrinkage performance.

### 3.2. Performance Tests of Cement Concrete

#### 3.2.1. Salt Freeze–Thaw Cycles Test

The specimens with the curing age of 24 days were immersed in 4% NaCl solution with the temperature of 20 ± 2 °C for 4 days to prepare for the salt freeze–thaw cycle test. Based on the fast freezing method in GB/T 50082-2009 [33], the test was carried out using a rapid freeze–thaw testing machine for concrete. The similar specimens of 100 mm × 100 mm × 400 mm were prepared in this experiment. The number of salt freeze–thaw cycles were 0, 25, 50 as well as 75 and one cycle took 2–4 h. During freezing and thawing, the lowest and highest temperatures of the specimen center were controlled within (−18 ± 2) °C and (5 ± 2) °C, respectively. The water level in the box was always at least 5 mm above the top of the specimens. The mass and dynamic elastic modulus of the specimens were measured after every 13 (12) freeze–thaw cycles. At the same time, in order to ensure the uniform destruction of the specimen and maintain the concentration of salt solution, the specimen was replaced up and down and the new salt solution was replaced. The flexural strength, compressive strength and chloride ion erosion depth were tested after each 25 cycles.

#### 3.2.2. Mass Loss and Dynamic Elastic Modulus Test

In the process of salt freeze–thaw cycles, the external damage of cement concrete was characterized by mass loss, which was caused by the spalling of specimen surface, and its development of internal microcracks could be reflected by the dynamic elastic modulus. Therefore, the mass loss rate and relative dynamic elastic modulus calculated via Equation (2) and Equation (3) were adopted to evaluate the external and internal damage degree of cement concrete after salt freeze–thaw cycles.

(2) $$\Delta W_{ni}=\frac{W_{0i}-W_{ni}}{W_{0i}}\times 100$$

where △*W_ni_* is the mass loss rate (%) of the *i*th concrete specimen after *n* freeze–thaw cycles, accurate to 0.01; *W*_0*i*_ is the mass (g) of the *i*th concrete specimen before salt freeze–thaw test; *W_ni_* is the mass (g) of the *i*th concrete specimen after *n* freeze–thaw cycles.

(3) $$P_{i}=\frac{{f_{ni}}^{2}}{{f_{0i}}^{2}}\times 100$$

where *P_i_* is the relative dynamic elastic modulus (%) of the *i*th concrete specimen after *n* freeze–thaw cycles; *f_ni_* is the transverse fundamental frequency (Hz) of the *i*th concrete specimen after *n* freeze–thaw cycles; *f*_0*i*_ is the initial value of transverse fundamental frequency (Hz) of the *i*th concrete specimen before salt freeze–thaw test.

#### 3.2.3. Flexural Strength and Compressive Strength Test

To investigate the damage of salt freeze–thaw cycles on flexural strength of cement concrete, four-point bending test was conducted using the universal testing machine. The upper and lower spans between supports were 100 mm and 300 mm, respectively. Loading control with the speed range of 0.05 MPa/s−0.08 MPa/s was set in the test. To better investigate the deterioration rule of pavement concrete after salt freeze–thaw cycles, the residual flexural strength was defined and calculated using the following Equation.

(4) $$L_{fi}=\frac{f_{0i}-f_{ni}}{f_{0i}}$$

where *L_fi_* is the residual flexural strength of the *i*th concrete specimen after *n* freeze–thaw cycles; *f*_0*i*_ is the initial value of flexural strength (MPa) of the *i*th concrete specimen before salt freeze–thaw test; *f_ni_* is the flexural strength (MPa) of the *i*th concrete specimen after *n* freeze–thaw cycles. *L_fi_* = 0 means there is not any damage and *L_f__i_* = 1 means unstable fracture occurred in the concrete specimen.

In order to ensure that the mechanical properties damage of cement concrete under coupling effect was evaluated more comprehensively, the compressive strength was also regarded as an indicator. According to JTG 3420-2020 [32], the compressive strength of the block was tested immediately after flexural strength test. Loading control in this test was set in the speed range of 0.5 MPa/s−0.8 MPa/s.

#### 3.2.4. Chloride Ion Erosion Depth Test

The resistance to chloride ion erosion of cement concrete was explored by studying the erosion depth of chloride ion in specimens after salt freeze–thaw cycles. On the basis of the AgNO_3_ chromogenic method of rapid chloride ion migration coefficient method (RCM) in GB/T 50082-2009 [33], the size of the specimen was φ100 mm × 50 mm cylinders. The specific steps are as follows: (1) the specimen was split into two semi-cylinders along the axial direction on the pressure testing machine and 0.1 mol/L AgNO_3_ solution color indicator was immediately sprayed on the split section. (2) After about 15 min, the section of the specimen was divided into 10 equal parts along its diameter and the penetration contour line should be traced by waterproof pen. (3) The distance between the color boundary and the bottom of the specimen was measured, ignoring the measuring points blocked by the aggregate. The final calculation of chloride erosion depth is shown in Equation (5).

(5) $$\bar{d_{n}}=\frac{1}{n}\sum_{i=1}^{n}d_{i}$$

where $\bar{d_{n}}$ is the average erosion depth (mm) after salt freeze–thaw cycles; *d_i_* is the erosion depth of the measuring point (mm); *n* is the total number of measuring points. (Each half of the test specimen was more than 5 values.)

### 3.3. Microscopic Test

#### 3.3.1. MIP test

The MIP test was conducted to investigate the pore structure characteristics of concrete using the PoreMaster-60 mercury injection instrument with the measurable pore size of 7 nm–100μm. The size of sample was about 1 cm×1 cm×1 cm.

#### 3.3.2. Hydration Characteristics Test

The internal structure of cement concrete might be affected by the early hydration process, which was most studied from the two aspects of hydration heat release curves and hydration degree. Considering the rapid progress of hydration reaction of cement in the first 3 days, the heat of hydration was constantly recorded by isothermal calorimeter on cement hydration heat tester to help characterize the hydration progress of cement. Hydration heat release rate and cumulative heat release quantity could be calculated with the guidance of the cement hydration heat test method (direct method) of GB/T 12959-2008 [34]. The test parameters were set as follows: temperature of the thermostatic water bath was 20 °C ± 0.1 °C; data acquisition interval was 30 min; total test time was 75 h; temperature range was 20–50 °C. The cement mortar was evenly mixed according to Table 3.

The hydration degree of concretes internally cured by SAP at final stage of curing age (day 28) and different times of salt freeze–thaw cycles could be evaluated by FTIR-960 Fourier transform infrared spectrometer. Scanning wave number range was 4000–400 cm^−1^ and resolution was 4.0 cm^−1^. Potassium bromide tabletting method was adopted to produce the observation samples.

#### 3.3.3. SEM

The resistance mechanism to salt freeze–thaw coupling effect of concrete internally cured by SAP was closely related to its microstructure, which would also help to explain the macroscopic properties. Thus, SEM was conducted to characterize the microstructure of cement concrete before and after salt freeze–thaw cycles. SU-1500 scanning electron microscope produced in Japan was used in this test, with the secondary electron resolution of 3.0 nm.

To more intuitively investigate the deterioration of concrete with the times of salt freeze–thaw cycles, the pores of the microstructure were quantitatively calculated via Image J software in this test.

## 4. Results and Discussions

### 4.1. Optimization of Internal Curing Parameters Based on Grey Target Decision Method

The flexural strength and compressive strength on day 28, shrinkage rate on day 3 and shrinkage interval length in the range of day 14 to day 28 were selected as evaluation indices. The specific experimental data are shown in Table 4. The target center of each test group was calculated by the grey target decision method to realize the optimization of SAP parameters, which laid a foundation for the study of the performance deterioration of SAP-concrete under the coupling effect of salt freeze–thaw.

The scheme could be optimized with the condition of uncertain weight information through the grey target theory applied in this paper. The target degree information of each scheme in the associated difference space was calculated and compared with the constructed standard model. The greater the targeting, the closer to the ideal solution.

Based on the results of the above mortar performance test, the grey target decision method calculation steps are as follows:

(1) Gray mode is constituted.

*w_i_* is defined as a multilevel indicator sequence and *w_i_*(*k*) is the date for indicator *k* of pattern *i*, which can be expressed as:

(6) $$w_{i}=[w_{i}(1),w_{i}(2),\ldots ,w_{i}(n)],\forall w_{i}(k)\in w_{i}\to k=1,2,\ldots ,n;\;i=1,2,\ldots ,m.$$

*w*(*k*) is the index pattern sequence, which can be given by:

(7) $$w(k)=[w_{1}(k),w_{2}(k),\ldots ,w_{m}(k)]$$

(2) A standard model is built.

The comprehensive performance of mortar is determined according to the maximum and minimum values of different indexes. Among them, flexural strength and compressive strength are endowed with the maximum polarity; on the contrary, shrinkage rate and shrinkage interval length are endowed with the minimum. Then the standard model is built as:

(8) $$w_{0}(1)=\max w_{i}(1)=\max[w_{1}(1),w_{2}(1),w_{3}(1),w_{4}(1),w_{5}(1),w_{6}(1),w_{7}(1),w_{8}(1)]$$

(3) Grey target transformation is conducted.

The target is defined as:

(9) $$Tw_{0}=x_{0}=(1,1,1,1),x_{i}(k)=\frac{\min[w_{i}(k),w_{0}(k)]}{\max[w_{i}(k),w_{0}(k)]}$$

The matrix *X* composed of (*x*_1_, *x*_2_, *x*_3_, *x*_4_)^T^ can be obtained from Equation (9), which is shown as:

$$X=\left[\begin{array}{ccccccccc} 1.000 & 0.929 & 0.988 & 0.893 & 0.952 & 0.881 & 0.917 & 0.838 & 0.929\\ 1.000 & 0.907 & 0.952 & 0.833 & 0.855 & 0.921 & 0.980 & 0.843 & 0.881\\ 0.493 & 0.844 & 0.909 & 0.829 & 0.936 & 0.892 & 0.836 & 0.797 & 1.000\\ 0.160 & 0.544 & 0.935 & 0.662 & 1.000 & 0.307 & 0.242 & 0.230 & 0.253 \end{array}\right]$$

(4) Grey relational difference information space is determined.

Difference information set is defined as:

(10) $$\Delta =(\Delta_{0}i(k)|\;i=1,2,3,4,\;k=1,2,3,4,5,6,7,8,9)$$

(11) $$\Delta_{01}(k)=[\Delta_{01}(1),\Delta_{01}(2),\Delta_{01}(3),\Delta_{01}(4),\Delta_{01}(5),\Delta_{01}(6),\Delta_{01}(7),\Delta_{01}(8),\Delta_{01}(9)]$$

(12) $$\Delta_{0i}(k)=|x_{0}(k)-x_{i}(k)|=|1-x_{i}(k)|$$

The matrix Δ composed of $(\Delta_{01},\;\Delta_{02},\;\Delta_{03},\;\Delta_{04},\;\Delta_{05},\;\Delta_{06},\;\Delta_{07},\;\Delta_{08}{)}^{T}$ can be calculated from Equation (10) to Equation (12), which is presented as:

$$\Delta =\left[\begin{array}{ccccccccc} 0.000 & 0.071 & 0.012 & 0.107 & 0.048 & 0.119 & 0.083 & 0.163 & 0.071\\ 0.000 & 0.093 & 0.048 & 0.167 & 0.145 & 0.079 & 0.020 & 0.157 & 0.119\\ 0.507 & 0.156 & 0.091 & 0.171 & 0.064 & 0.018 & 0.164 & 0.203 & 0.000\\ 0.840 & 0.456 & 0.065 & 0.338 & 0.000 & 0.693 & 0.758 & 0.770 & 0.747 \end{array}\right]$$

Thus, it can be obtained that:

(13) $$\underset{i}{\max}\underset{k}{\max}\Delta_{0i}(k)=0.840,\;\underset{i}{\max}\underset{k}{\max}\Delta_{0i}(k)=0$$

(5) Target coefficient is calculated.

Target coefficient *Y* is defined as:

(14) $$Y(x_{0}(k),x_{i}(k))=\frac{\underset{i}{\min}\underset{k}{\min}\Delta_{0i}(k)+0.5\underset{i}{\max}\underset{k}{\max}\Delta_{0i}(k)}{\Delta_{0i}(k)+0.5\underset{i}{\max}\underset{k}{\max}\Delta_{0i}(k)}$$

Based on the matrix Δ and the Equations (13) and (14), the matrix *Y* is got as:

$$Y=\left[\begin{array}{ccccccccc} 1.000 & 0.855 & 0.972 & 0.797 & 0.897 & 0.779 & 0.834 & 0.721 & 0.855\\ 1.000 & 0.819 & 0.898 & 0.716 & 0.743 & 0.842 & 0.954 & 0.728 & 0.779\\ 0.453 & 0.729 & 0.822 & 0.711 & 0.867 & 0.795 & 0.720 & 0.675 & 1.000\\ 0.333 & 0.480 & 0.866 & 0.554 & 1.000 & 0.377 & 0.356 & 0.353 & 0.360 \end{array}\right]$$

(6) The weight coefficient is determined by the entropy weight method.

In this part, regarding the X decision matrix as a risk assessment matrix, the normalized processing of data is carried out. The characteristic proportion of the *i*th model on the *k*th evaluation index is calculated as:

(15) $$f_{k}(i)=\frac{x_{i}(k)}{\sum_{i=1}^{9}x_{i}(k)}$$

Information entropy *H_k_* of index *k* is calculated as:

(16) $$H_{k}=-\frac{1}{\ln9}\sum_{i=1}^{9}f_{k}(i)\ln f_{k}(i)$$

Objective weight *α_k_* of each index is obtained using entropy as:

(17) $$\alpha_{k}=\frac{1-H_{k}}{\sum_{k=1}^{4}\left(1-H_{k}\right)}$$

Results of weight are as:

$$\alpha_{k}=(0.256,\;0.255,\;0.254,\;0.235)$$

Finally, the calculation results of the target center are shown in Table 5.

From the above table, it can be seen that the order of mortar target degree from highest to lowest was SAP-1-0.15%, WRA-1-0.15%, WRA-2-0.15%, SAP-1-0.10%, SAP-2-0.15%, SAP-2-0.10%, control group, SAP-1-0.20% and SAP-2-0.20%. The control group was ranked in 7th position, indicating that the addition of SAP could balance well the relationship between shrinkage performance and mechanical properties of cement-based materials; thus, the better curing effect was achieved.

According to the experimental results, it can be found that the performance of SAP-mortar with the particle size of 100 mesh was better than 200 mesh with the same SAP dosage. It could be analyzed that the water conservation ability of 100 mesh is more powerful, for its liquid absorption rate is slightly higher than 200 mesh. During the curing process, SAP gels of 100 mesh particle size can release more internal curing water, which may expand the region of curing and promote the hydration of cement, and thereby, the performance of SAP-mortar is improved. As for the curing effect of SAP dosage, the order of SAP-mortar performance from large to small was 0.15%, 0.10% and 0.20% with the same SAP particle size, whether it was 100 mesh or 200 mesh. This phenomenon may also relate to the water conservation ability. There are more particles in the SAP dosage of 0.15% than 0.10%, which will absorb more internal curing water; therefore, a better curing effect is achieved.

There is no doubt that the curing effect will improve with the increase of dosage based on the above analysis, but it was interesting to note that the mortar internally cured by SAP dosage of 0.20% was inferior to the control group. This is because it is easy for the phenomenon of ‘micro-agglomeration’ to occur during the progress of cement mortar mixing, which leads to the hindering of internal curing moisture release and the weakening of the curing effect, and in this case, the target degree of the mortar was reduced.

It can be known from the test results that the use of water-resisting agent could reduce the shrinkage of the mortar to some extent, but the mechanical properties were not improved, which was reflected in the fact that the target degree of SAP-1-0.15% was greater than that of WRA-1-0.15%. The above results could be interpreted as showing that water-resisting agent may reduce the loss of the inside water of the mortar and increase the internal humidity, and consequently, the plastic shrinkage caused by hydration water consumption and water evaporation of the cement itself is restrained. However, the humidity of mortar in a certain range from the surface layer decreases due to the lack of sprinkling curing, and it may promote the generation of drying shrinkage microcracks, which has a negative impact on the mechanical properties.

In summary, the optimized results of SAP particle size, dosage and curing condition were 100 mesh, 0.15% and natural curing. The results showed that SAP has an optimal dosage, which was consistent with the findings in the literature [11]. Considering that SAP dosage would have an important impact on the effect of curing and cement mortar could only reflect the performance of cement concrete to a certain extent, the SAP dosage of 0.125% and 0.175% were also selected as variables in curing cement concrete to find the optimal SAP dosage more accurately. In the test of cement concrete, water reducer was adjusted according to the requirements of slump pavement concrete. The specific design of internal curing pavement concrete mix proportion is shown in Table 6.

### 4.2. Deterioration Analysis of Salt Freeze–Thaw Cycles of Pavement Concrete

#### 4.2.1. Mass Loss Rate and Relative Dynamic Elastic Modulus

**Figure 3:**
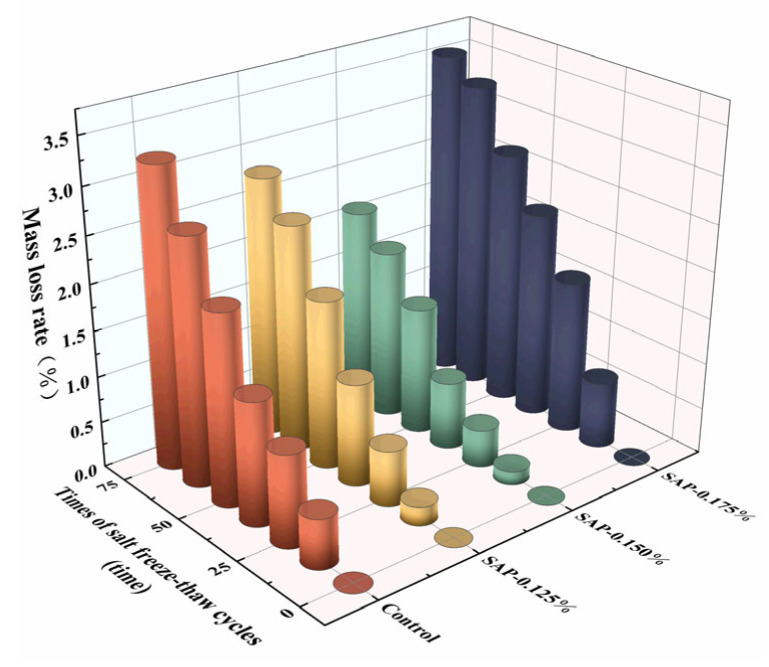
Mass loss rate of pavement concretes with different times of salt freeze–thaw cycles.

**Figure 4:**
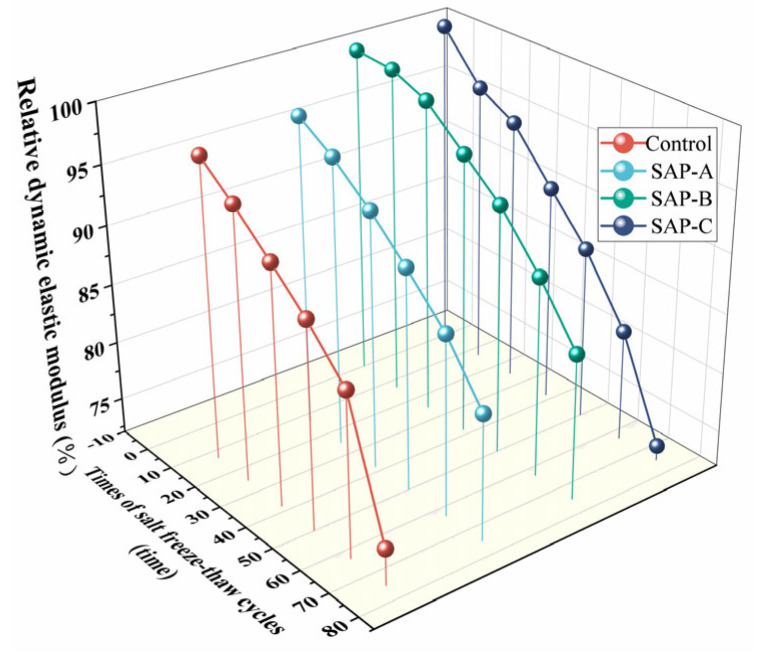
Relative dynamic elastic modulus of pavement concretes with different times of salt freeze–thaw cycles.

The mass loss rate and relative dynamic elastic modulus of test groups of different salt freeze–thaw cycle times are shown in Figure 3 and Figure 4, respectively.

From Figure 3, it can be observed that the mass loss rates of SAP-0.125% and SAP-0.150% internal curing groups were always lower than that of the control group during the salt freeze–thaw cycles. When the cycle reached 75 times, the mass loss rates of SAP-0.125% and SAP-0.150% were 86.83% and 65.76% of the control group, respectively. As for the relative dynamic elastic modulus (see Figure 4), the values of SAP-0.125% and SAP-0.150% were 1.10 times and 1.12 times that of the control group. The above results indicated that salt freeze–thaw resistance of the concrete internally cured with 0.125% and 0.150% SAP was enhanced and the improvement effect of the latter was better than the former. The reason for the damage to the cement concrete could be mainly explained by the hydrostatic pressure. The volume of pores inside the concrete are increased by 9% when the water freezes in the pores, which may result in the expansion stress exceeding the critical value, leading to the appearance of cracks and the destruction of pore structure inside the specimen. Consequently, the spalling of cement paste and aggregate on the surface of the specimen increases and the damage to the cement concrete is aggravated. The frost-heaving resistance of SAP could be attributed to the fact that the in situ residual holes formed by SAP gels are mostly closed holes [8]. Those closed holes with a certain elastic deformation ability can play a role of air-entraining agent to provide some buffer space for expansion stress caused by water freezing. The greater the SAP dosage, the more buffer space can be produced; thus, the salt freeze–thaw resistance of SAP-0.150% is better than SAP-0.125%.

Nevertheless, the salt freeze–thaw resistance of SAP-concrete did not necessarily improve with the increase of SAP dosage. It is difficult to ignore the phenomenon that in the concrete internally cured by 0.175% SAP, the mass loss rate was higher than the control group and the relative dynamic elastic modulus was lower than the control group during the salt freeze–thaw cycles. It is speculated that there are more SAP pores remaining in the cement concrete cured with a higher SAP dosage, which is detrimental to salt freeze–thaw resistance. However, in the last 25 cycles, the mass loss rate of the control group was 2.71 times that of the SAP-0.175%. At the same time, the decrease of relative dynamic elastic modulus in the control group was 10.77% while it was only 4.57% in SAP-0.175%. The above performance demonstrated that the effect of salt freeze–thaw resistance of SAP-0.175% was gradually improved with the extension of the cycles. Based on the above results, it can be speculated that the SAP gels can absorb salt solution and hinder further penetration of chloride ions, and therefore, the deterioration of the cement concrete was eased.

#### 4.2.2. Flexural Strength and Compressive Strength

**Figure 5:**
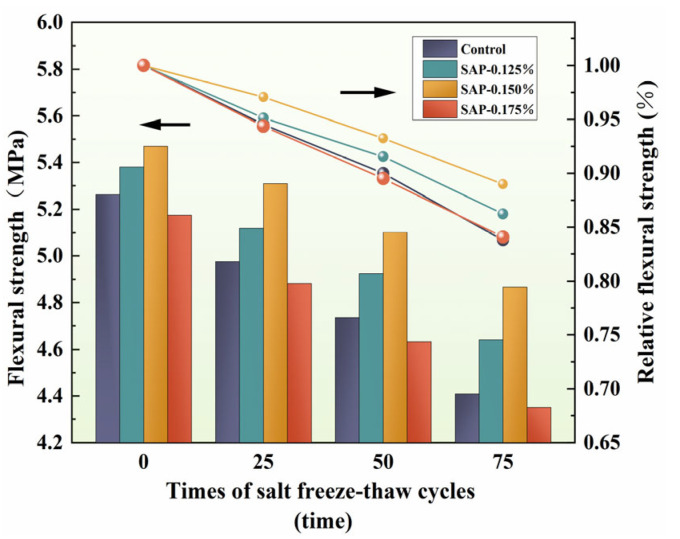
Variation of flexural strength and relative flexural strength with salt freeze–thaw cycles times.

The flexural strength and relative flexural strength of cement concrete under different salt freeze–thaw cycles times are described in Figure 5.

As seen in Figure 5, the flexural strength and relative flexural strength of SAP-0.125% and SAP-0.150% were always higher than the control group. At the later stage of the salt freeze–thaw cycles (50–75 times), the slope of relative flexural strength broken-line of SAP-concrete was smaller than that of the control group, which meant less loss of flexural strength. After 75 cycles, the flexural strength of the control group, SAP-0.125%, SAP-0.150% and SAP-0.175% decreased by 16.21%, 13.76%, 11.02% and 15.91%, respectively, compared with the specimens before the salt freeze–thaw test. Among them, the flexural strength of SAP-0.125% and SAP-0.150% were 5.24% and 10.39% higher than the control group. Combined with the influencing factors of flexural strength, the damage reduction of SAP-0.125% and SAP-0.150% after salt freeze–thaw cycles is owed to the decrease of microcracks inside the concrete, and the increase of the adhesion of cement stone and aggregate attributed to internal curing. The curing range of 0.150% SAP dosage is larger than that of 0.125% so the effect of SAP-0.150% is more obvious.

It could also be observed that the flexural strength of the concrete internally cured by the 0.175% SAP was always lower than that of the control group before and after the test. The flexural strength reduction of SAP-0.175% could be explained by the following two aspects. On the one hand, more honeycomb holes are formed with the more dosage of SAP when the SAP has released water and returns to a dry state. At the same time, the liquid water gradually froze in the freezing stage, and the volume increased, resulting in more cracks [35]. Therefore, the stress concentration occurred at the holes when the specimen was subjected to flexural stress, which may have further promoted the initiation of internal microcracks and exacerbated its expansion. On the other hand, the strength loss caused by excessive residual holes generated by SAP gels is difficult to balance with the effect of hydration filling enhancement. Moreover, the line of relative flexural strength between SAP-0.175% and the control group were basically coincident during the salt freeze–thaw cycles, which indicated that the damage degree of the flexural strength of SAP-0.175% was not exacerbated, compared with the control group.

**Figure 6:**
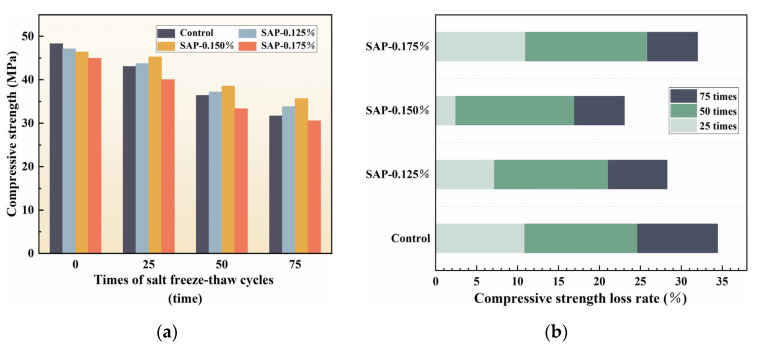
Variation of compressive strength and compressive strength loss rate with salt freeze–thaw cycles times: (**a**) Compressive strength; (**b**) Compressive strength rate.

To further explore the effect of SAP dosage on the mechanical properties of cement concrete during salt freeze–thaw cycles, the compressive strength of the test block was measured after the flexural strength test was carried out. The compressive strength and its loss rate are presented in Figure 6.

According to Figure 6, the compressive strength of the control group was greater than that of SAP-concrete at the end of curing age, which might be caused by the existence of residual pores formed by the SAP gels. However, the control group gradually lost its advantage under the coupling effect of salt freeze–thaw cycles and its compressive strength was exceeded by SAP-0.125% and SAP-0.150% from 25 cycles. After 75 cycles, the compressive strength of SAP-0.125% and SAP-0.150% increased by 6.7% and 12.67%, respectively, compared with the control group. The compressive strength loss rates of SAP-0.125% and SAP-0.150% were 28.27% and 23.06%, while it was as high as 34.43% in the control group. The analysis of the compressive strength improvement in the above SAP-concrete is that the addition of SAP can promote the hydration degree and more hydration products like C-H-S and Ca(OH)_2_ are generated to fill the pores, thus, the internal structure of the cement concrete is compacted. The more hydration products may be produced with the a higher dosage of SAP, resulting in the better salt freeze–thaw resistance in SAP-0.150% than SAP-0.125%.

Unlike the representation of the above SAP-concrete, the compressive strength of SAP-0.175% was always lower than the control group before and after salt freeze–thaw cycles. The reason here is similar to the analysis in flexural strength. The concentrated stress generated at the holes affects the compressive strength of the concrete when the specimen is compressed. Otherwise, the compressive strength loss rate of SAP-0.175% was almost the same as the control group during the progress of test, which was 31.99% in 75 cycles and was 2.44% lower than the control group. The result shows that salt freeze–thaw cycles did not aggravate the damage to the compressive strength of concrete internally cured by high SAP dosage, which was the same as the experimental results of flexural strength.

#### 4.2.3. Depth of Chloride Ion Erosion

**Figure 7:**
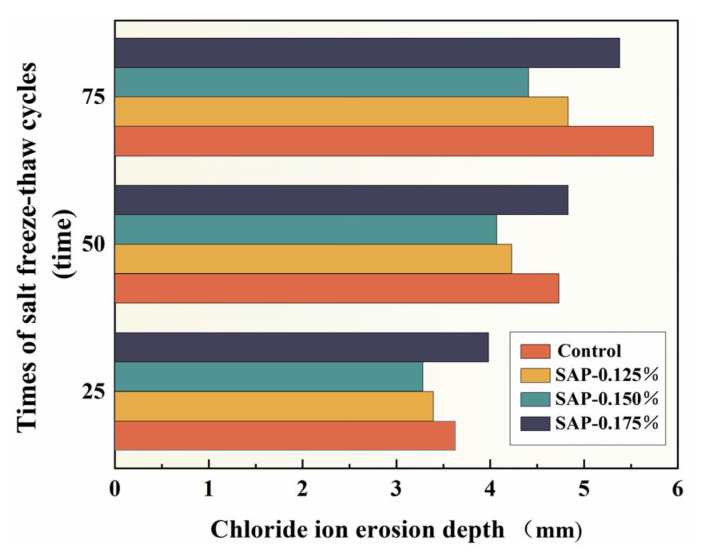
Variation of chloride ion erosion depth with salt freeze–thaw cycles times.

Figure 7 illustrates the variation of chloride ion erosion depth with salt freeze–thaw cycles times.

Figure 7 reveals that the chloride ion erosion depth of SAP-concrete decreased first and then increased with the increment of SAP dosage during salt freeze–thaw cycles. In addition, the depths of chloride ion erosion of SAP-0.125% and SAP-0.150% were less than the control group in the progress of the test and they were 15.85% and 23.18% lower than the control group after 75 cycles, respectively. The enhancement of chloride ion erosion resistance with the appropriate SAP dosage could be summarized as follows: (1) the formation of closed pores and reduction of microcracks inside the concrete attributed to SAP curing might block the channels of chloride migration; and (2) the adsorption of salt solution by SAP gels also hinders the further penetration of chloride ions. The distribution of SAP dosage of 0.150% is wider than that of 0.125%, which can carry out the hydration reaction in more areas to reach a better internal curing effect.

Although the chloride ion erosion depth of SAP-0.175% was greater than the control group in the first 50 salt freeze–thaw cycles, it was 6.28% lower than that of the control group at 75 times. This phenomenon might be related to the lower cement matrix density and higher microstructure connectivity (higher porosity) of the concrete with the relatively high SAP dosage; as a result, more opportunities for external corrosive ions to invade and transfer into the concrete interior are provided [36].

### 4.3. Damage Prediction Model under Salt Freeze–Thaw Coupling Effect

The external destruction of concrete might be caused by the internal damage, which is comparatively difficult to measure directly. To better reflect the damage decay inside the concrete, the damage degree of the specimen is defined by the relative dynamic elastic modulus according to the Equation (18).

(18) $$D=1-\frac{E_{n}}{E_{0}}$$

where *D* is the damage degree; *E*_0_ is the initial dynamic elastic modulus of concrete before salt freeze–thaw test; *E_n_* is the dynamic elastic modulus of concrete after *n* freeze–thaw cycles.

**Figure 8:**
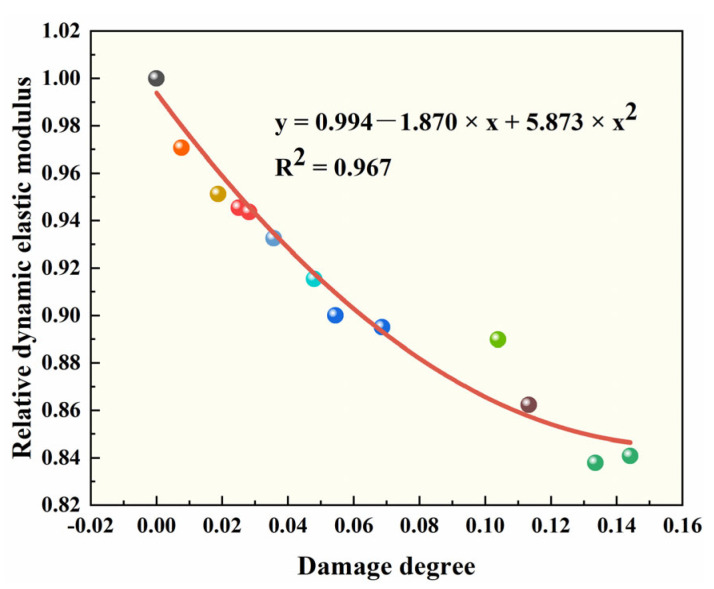
Fitting analysis of relative dynamic elastic modulus and damage degree.

Considering that the flexural strength is an important index to evaluate the performance of pavement concrete, the fitting of the damage degree and relative flexural strength of the specimens was conducted, as shown in Figure 8.

From the curve in Figure 8, it can be seen that the relative flexural strength decreased with the increase of the damage degree. The mathematical relationship between the two is an upward opening quadratic function with the correlation coefficient of 0.967. The rationality and reliability of the model were verified, and accordingly, the relative flexural strength of the concrete after salt freeze–thaw cycles could be evaluated by nondestructive and easily measured index of damage degree.

### 4.4. Mechanism of Salt Freeze–Thaw Resistance

#### 4.4.1. Analysis of Pore Structure Characteristics

**Figure 9:**
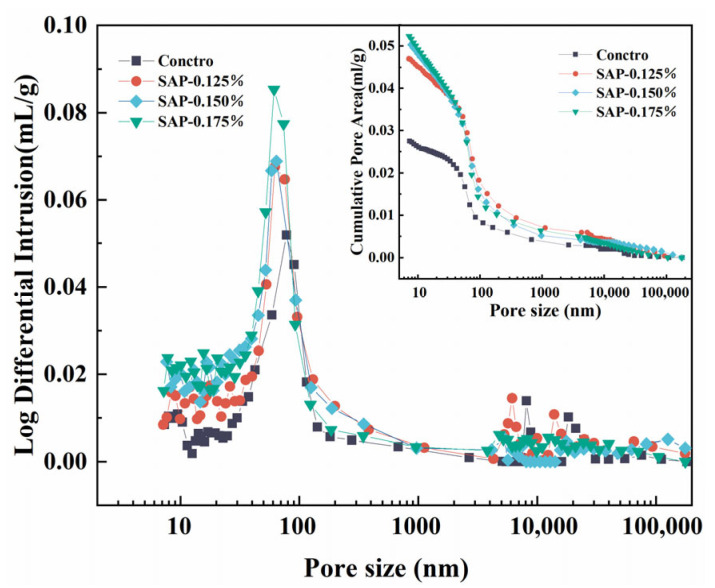
Curve of pore size distribution of pavement concrete before salt freeze–thaw test.

An MIP test was carried out to investigate the pore structure between the control group and SAP-concrete. Figure 9 presents the curve of pore size distribution, and at the same time, the total surface area and average pore size is shown in Table 7.

As is described in Figure 9, the curve of the SAP-concrete moved up significantly and the porosity enlarged with the increase of SAP dosage, compared with the control group. The increase in porosity might be detrimental to the strength of the concrete, as reflected in the fact that the compressive strength of the SAP-concrete was lower than that of the control group at the end of the curing age. Combined with the value of the total surface area illustrated in Table 7, it was suggested that the degree of SAP-0.175% increased the most, followed by SAP-0.150% as well as SAP-0.125%. The larger the total pore area is, the greater the number of residual pores left by SAP gels, which confirms that more buffer space could be provided in SAP-0.150% than SAP-0.125% during the progress of frost. Nevertheless, porosity does not always have a positive impact for the resistance of salt freeze–thaw cycles of concrete. It has been found in the correlative study that with the increase of freeze–thaw cycles, the small pores inside the concrete might be transformed into large pores, and in this case, the proportion of harmless pores decreased and the proportion of multi-hazard pores increased [37]. That is why SAP-0.175% displayed a poor performance in the macroscopic test.

From the point of view of pore size, it can be discovered that the total pore area of the internal curing group was higher than that of the control group, while the average pore size of SAP-0.125%, SAP-0.150% and SAP-0.175% were 95.66%, 80.57% and 78.53% of the control group, which implied that the addition of SAP could refine the pore size. It can be known based on the osmotic pressure theory that the freezing point of the solution in pores grows with the increase of pore diameter. Particularly, salt solution has high moisture absorption and water retention, which would greatly improve the pore saturation, accelerating salt freeze–thaw damage. Meanwhile, the peak value of the pore size distribution curve of the SAP-concrete moved forward, compared with the control group. It could be interpreted that the SAP internal curing may reduce the most probable pore size of cement concrete (the pore size corresponding to the peak value of the differential curve) and connected pores cannot be formed when the pore size is smaller than that. The above analysis demonstrates that the addition of SAP can improve the pore structure of cement concrete to resist the salt freeze–thaw.

**Figure 10:**
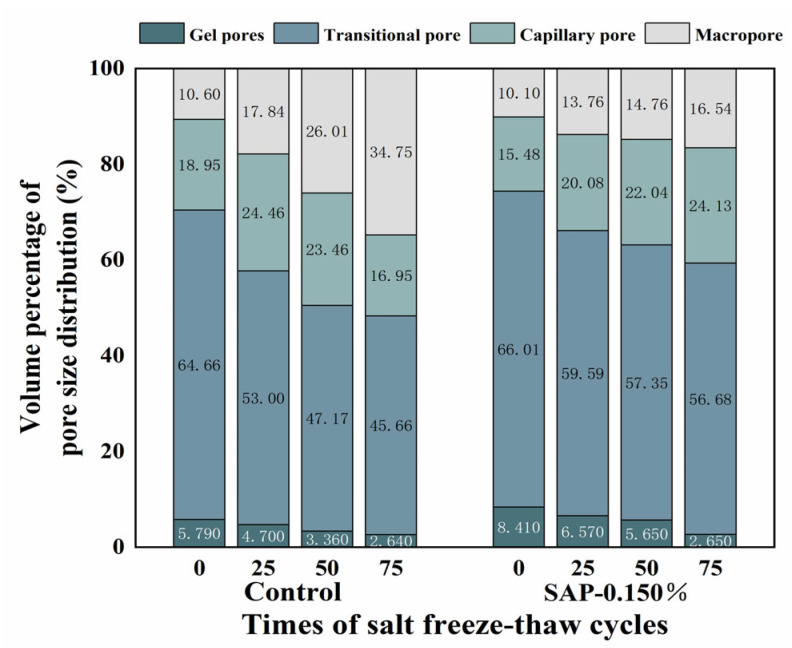
Cumulative pore volume percentage with salt freeze–thaw cycles times.

To find the influence of SAP internal curing on the pores inside cement concrete during salt freeze–thaw process, the control group and SAP-0.150% (the optimal group in macroscopic performance) were selected. The cumulative pore volume percentages are shown in Figure 10, which are classified according to the principles of gel pores (<10 nm), transitional pores (10–100 nm), capillary pores (100–1000 nm) and macro pores (>1000 nm).

As seen in Figure 10, concrete mixed with or without SAP was mainly composed of transitional pores. The sum percentage of transition pores and gel pores of SAP-0.150% increased by 3.97% compared with the control group before testing, which would play a role of ‘air-entraining antifreeze’ in the salt freeze–thaw cycles. During the progress of testing, the percentage of transition pores and gel pores in SAP-0.150% was markedly greater than that of the control group; in the meantime, the contents of macro pores and capillary pores were reduced. Generally, the generation of macro pores and capillary pores dominate the process of concrete exposed to NaCl solution. After 75 cycles, the macro pores and capillary pores of SAP-0.150% decreased by 11.03% compared with the control group. This indicates that SAP internal curing could improve the pore structure under the coupling effect of salt freeze–thaw.

#### 4.4.2. Analysis of Hydration Characteristics

**Figure 11:**
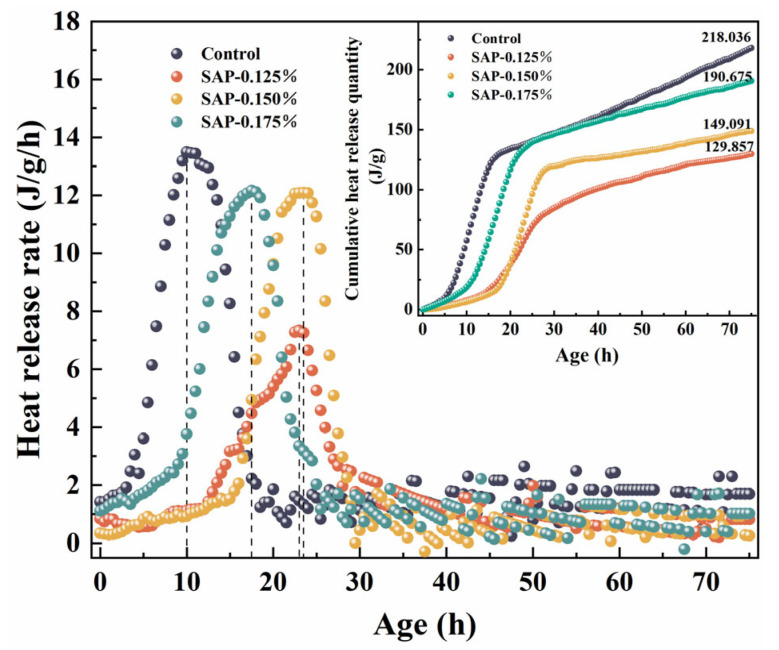
Hydration heat release curves: heat release rate and cumulative heat release quantity.

The variation curves of the hydration heat release rate and the cumulative heat release quantity of mortars with SAPs of different dosage are presented in Figure 11.

It is clear in Figure 11 that not only the values of cumulative heat release quantity in 75 h of mortars internally cured by SAP reduced significantly, but also the duration of the hydration induction period was prolonged and the appearance of the exothermic peak was delayed. Correspondingly, the adding of SAP could decrease the peak values of heat release rate for mortars. The analysis of the phenomena contains two aspects: firstly, the heat-transfer capability of SAP-mortars is lower than the control group since several residual pores were formed by SAP gels. Secondly, SAP gels absorbed part of heat during the hydration stage, which can slow down the early hydration of the cement. The above hydration characteristics of the SAP internal curing will effectively suppress the microcracks caused by temperature deformation in the early stage.

Figure 11 exhibits the heat release rate of the control group, SAP-0.125%, SAP-0.150% and SAP-0.175%, peaked at 10 h, 23 h, 23.5 h and 17.5 h, respectively, with the peak values of 13.462 J·g^−1^·h^−1^, 7.333 J·g^−1^·h^−1^, 12.091 J·g^−1^·h^−1^ and 12.164 J·g^−1^·h^−1^. It could be concluded that the peak time of hydration heat release rate moved forward, and the peak value increased in the internal curing group with the increase of SAP dosage. This is because the actual amount of internal curing water absorbed by SAPs was less than the theoretical values, resulting in the internal curing water participating in the hydration of cement in the form of mixed water, which increased the hydration rate. Among the internal curing group, SAP-0.175% had the largest contribution to the water of the hydration reaction system for it had the most additional water diversion. Accordingly, its hydration heat release rate peak appeared earliest with the largest peak.

Figure 11 also shows that the ranking of cumulative heat release quantity of mortars from highest to lowest was the control group (218.036 J·g^−1^), SAP-0.175% (190.675 J·g^−1^), SAP-0.150% (149.091 J·g^−1^) and SAP-0.125% (129.857 J·g^−1^). The early hydration degree of cement-based materials could be characterized by the cumulative heat release quantity to a large extent. Obviously, the control group had the highest degree of hydration at 75 h among the test groups. As for the internal curing group, hydration degree rose with the increase of the SAP dosage. This is because more SAP dosage is widely and uniformly distributed in the mortars and more intensive hydration reaction can be carried out, thus, the degree of hydration is improved.

Given the above results, although the early hydration degree of SAP-0.175% was the highest in the internal curing group, it was not conducive to reducing microcracks caused by temperature deformation because of the early arrival of the exothermic rate and higher peak value. Oppositely, SAP-0.125% greatly reduced the peak heat release rate, but its early hydration degree was the lowest in the test group. In summary, SAP-0.150% could better balance the relationship between the time as well as the value of peak heat release rate and early hydration degree, which might better inhibit early temperature shrinkage cracks.

**Figure 12:**
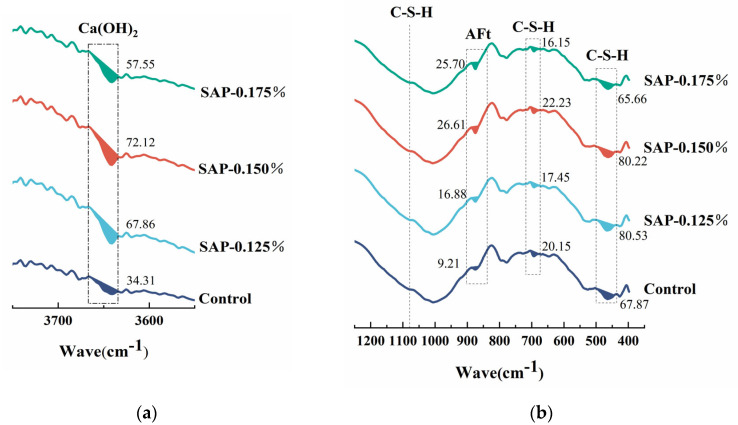
FTIR spectrum of pavement concrete with different SAP dosage after 75 cycles: (**a**) the peak area of Ca(OH)_2;_ (**b**) the peak area of C-S-H and AFt.

To better understand the hydration degree of internal curing concrete, the comparison of the FTIR spectrum of the control group and SAP-concrete after 75 cycles is depicted in Figure 12. A qualitative analysis method was used to analyze FTIR spectra in most existing literature [38]. For the purpose of obtaining a more intuitive change variation of each component, the absorption peak area was quantitatively analyzed in this paper, so that the peak area was often more accurate than the peak height.

It can be observed from Figure 12 that the characteristic peak of Ca(OH)_2_ (about 3642 cm^−1^) of SAP-concrete was greater than that of the control group, and its content could directly reflect the hydration degree of pavement concrete. The Ca(OH)_2_ absorption peaks of SAP-0.125% and SAP-0.150% were close, and both were higher than that of SAP-0.175%, denoting that the curing effect of the two former was better. In particular, the peak area of Ca(OH)_2_ in SAP-0.150% was 2.10 times that of the control group, which could also help explaining that it had the best macroscopic performance after 75 cycles. The peaks of 1081 cm^−1^ as well as 1024 cm^−1^, 692 cm^−1^ and 463 cm^−1^ correspond to the characteristic peaks of C-S-H gel, which represented the main hydration products and cementitious materials of Portland cement. Compared with the peaks of 1081 cm^−1^ as well as 1024 cm^−1^, the characteristic peaks of C-S-H gel at 692 cm^−1^ and 463 cm^−1^ changed obviously, hence, the two peak areas were quantified. The sum of the small peak areas of SAP-0.125% and SAP-0.150% at these two sites was greater than that of the control group, signifying a higher degree of polymerization of the silicate grid. Furthermore, ettringite is also an important hydration product of cement, which is related to the characteristic peak of AFt or AFm (about 874 cm^−1^). It can be found that the peak areas of AFt or AFm in SAP-concrete were increased compared to the control group. It is worth noting that the peak areas of AFt or AFm of SAP-0.150% were 2.89 times that of the control group, indicating that the amount of hydration product could be increased to some extent with the internal curing of an appropriate amount of SAP.

**Figure 13:**
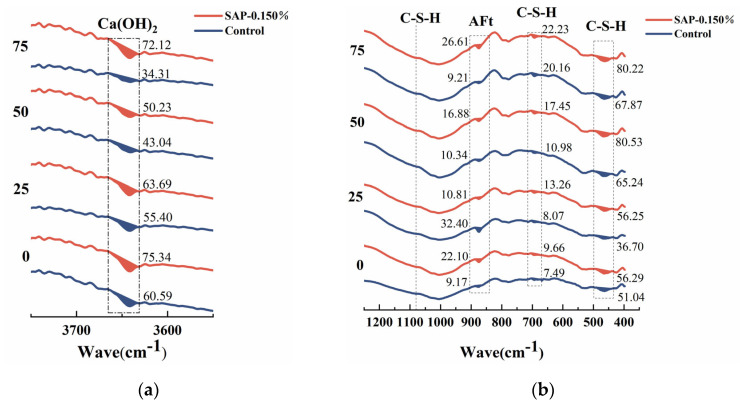
FTIR spectrum of SAP-0.150% and the control group with different times of salt freeze–thaw cycles: (**a**) the peak area of Ca(OH)_2;_ (**b**) the peak area of C-S-H and AFt.

In order to clarify the effect of SAP on cement hydration during salt freeze–thaw cycles, the FTIR spectrum of SAP-0.150% and the control group was selected for comparison, as shown in Figure 13.

From Figure 13, it is identified that the peak area of Ca(OH)_2_ of SAP-0.150% was larger than the control group before testing, indicating that the hydration degree at the end of the curing age was promoted, contributed by SAP internal curing. In the progress of the salt freeze–thaw cycles, the absorption peak area of Ca(OH)_2_ in the control group gradually decreased, for the reason that the NaCl solution reacted with Ca(OH)_2_ to form a salt expansion product. Notably, the peak area of Ca(OH)_2_ of SAP-0.150% decreased first and then increased, which reached the minimum value at 50 cycles. It could be speculated that SAP can play a role of ‘re-absorption’, that is to say, SAP has released water and turn back to dry state at the end of curing period, and it will continue to absorb water in the early stage of salt freeze–thaw testing. In the later stage of the test, the internal curing water can be gradually released, whereby, the secondary hydration of the unhydrated cement is promoted, consuming part of Ca(OH)_2_, and the content of it degrades. The corresponding macroscopic test results showed that the performance damage degree of the internal curing group was lower than the control group in 50 to 75 cycles. In the meantime, it can be seen that during the process of the salt freeze–thaw cycles, the sum of the C-S-H absorption peak areas at 463 cm^−1^ and 692 cm^−1^ of the internal curing group was larger than that of the control group, indicating that the amount of hydration product was more, which might produce a denser internal structure.

#### 4.4.3. Analysis of Microstructure

The deterioration law of macroscopic performance can be better explained with the aid of microscopic morphology; furthermore, the effect of SAP addition on the salt freeze–thaw resistance of cement concrete can be analyzed. Through macroscopic experiments, it was found that the SAP internal curing effect with the dosage of 0.150% was more significant. In order to further study its influence mechanism, SEM technology was used to observe the microstructure of the cement matrix of the control group and SAP-0.150% internal curing group before and after the salt freeze–thaw cycles.

**Figure 14:**
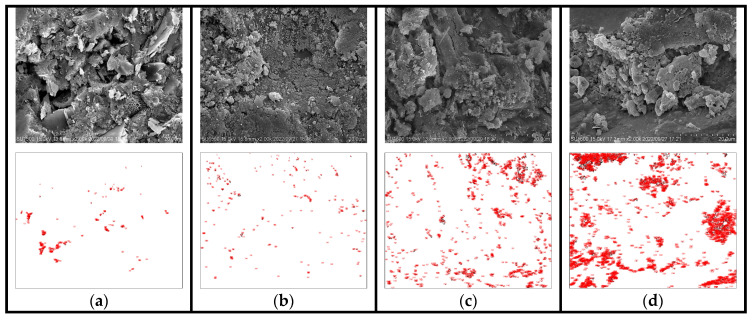
Micromorphology and the correlating pore outline drawing of the control group with different cycles times: (**a**) 0, (**b**) 25, (**c**) 50, (**d**) 75.

**Figure 15:**
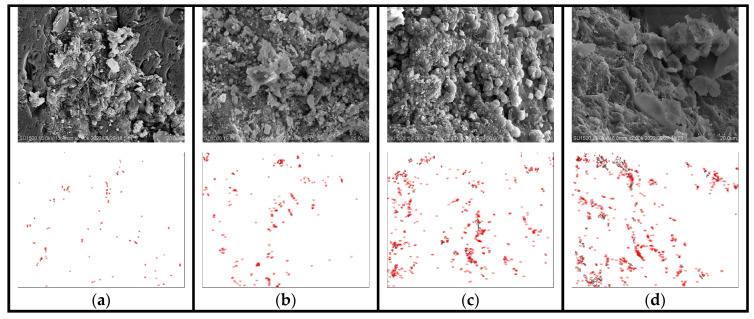
Micromorphology and the correlating pore outline drawing of SAP-0.150% with different cycles times: (**a**) 0, (**b**) 25, (**c**) 50, (**d**) 75.

Furthermore, as for the quantitative analysis of microscopic morphology, the existing literature has tried to measure the pore size [39]. In this paper, Image J software was used to analyze the correlating porosity, which would help to quantitatively characterize the deterioration of the concrete. Figure 14 and Figure 15 present the micromorphology and the correlating pore outline drawings of the control group and SAP-0.150%. Table 8 presents the statistical summary diagram of the pores; particularly, the % area means porosity, which is the ratio of pore area to micromorphology area.

Compared with the microstructure of the internal curing group (Figure 15a) before testing, it can be seen that the overall structure of the control group (Figure 14a) was relatively loose, and the amount of hydration product was less. Additionally, the combination degree of hydration products was low, and visible pores appeared in the control group, which might provide more channels for chloride ions to invade, accelerating the damage of the concrete. Furthermore, as shown in Figure 14b,c, the cracks appeared in 25 cycles, which developed into larger cracks in 50 cycles. This is due to the fact that those cracks may be filled with salt solution during the ice thawing stage, increasing the humidity inside the material. In this case, the greater expansion stress inside the concrete is produced, which aggravates the damage of salt expansion in the concrete.

It can also be recognized from Table 8 that with the increase of cycles, the count of pores and porosity increased obviously. Particularly, the porosity of the control group increased by 1.659% during the cycle times of 50 to 75. That is why the performance damage of the control group exacerbated in the progress of the last 25 cycles. Suffering 75 cycles, the overall structure was incompact with the dense pores on the matrix in the control group (see Figure 14d). Furthermore, the count of pores, total area and porosity of the control group reached 3152 pixel ², 17,434 pixel ² and 2.715%.

Table 8 reveals that the total area of pores and the porosity of the internal curing group was slightly higher than the control group before testing, which also verified that the SAP adding might introduce pores. In addition, as presented in Figure 15a, the amount and compaction of hydration products were both apparently increased, which could well fill the residual pores formed by SAP gels. This meant that the relationship between them was preferably balanced. Moreover, the tight combination between hydration products and pore boundary was realized, contributing to a denser internal structure. After 25 cycles, it can be seen that some hydration products in the pores reduced in Figure 15b. During the salt freeze–thaw process, although the internal structure of SAP-concrete gradually loosened, no obvious cracks and holes were observed. Meanwhile, the increase in the count of pores and porosity was smaller than that of the control group. Even after 75 cycles, only some pores were generated, shown in Figure 15d, and most of them were tiny pores. The count of pores and porosity in SAP-0.150% was 22.72% and 46.04% of the control group, respectively. The above results proved that the salt freeze–thaw resistance was enhanced by SAP internal curing again.

To better study the relationship between SAP dosage and internal curing effect, the parameters of the pores of the pavement concrete after 75 cycles were compared. The calculation results are shown in Table 9.

It can be seen from Table 9 that with the increase of SAP dosage, the count and % area of pores increased first and then decreased after 75 cycles. The internal curing effect of SAP-0.150% dosage was the best among the test groups, followed by the SAP-concrete internally cured by 0.125% SAP. The count and % area of pores in SAP-0.125% were 86% and 57% of the control group, respectively. This indicated that the pore parameters of SAP-concrete under the curing of 0.125% were improved, but the effect was not as good as SAP-0.150%. The reason is that SAP with low dosage may have the problem of insufficient curing. However, the count of pores in SAP-0.175% was 1.85 times that of the control group, signifying that a high dosage of SAP may leave more pores after water release, which is not conducive for concrete to resist salt freeze–thaw cycles. In summary, moderate SAP dosage can achieve better curing effect, which is consistent with the conclusions of the previous macro and micro test results.

## 5. Conclusions

The aim of this research was to optimize the SAP parameters and investigate the performance deterioration as well as the improving mechanism of the concrete internally cured by SAP under the coupling effect of salt freeze–thaw. The conclusions are summarized as follows:

1. The particle size, dosage and curing condition of SAP were optimized as 100 mesh, 0.150% and natural curing based on the grey target decision method via the mortar test.
2. The reduction in mass loss rate and relative dynamic elastic modulus were improved and the resistance to chloride ion erosion was enhanced by SAP internal curing. After 75 cycles, the mass loss rate and the relative dynamic elastic modulus of SAP-0.150% were 65.76% and 1.12 times of the control group, respectively. Furthermore, the chloride ion erosion depth of SAP-0.150% was 23.18% lower than the control group.
3. The strength of the specimen improved more significantly as the cycles of salt freeze–thaw increased. The mathematical relationship between relative flexural strength and damage degree was an upward opening quadratic function.
4. The influence mechanism of the salt freeze–thaw resistance of SAP internal curing was as follows: Firstly, SAP refined the pore structure. Secondly, the microcracks were decreased with the effect of SAP internal curing. Finally, the hydration degree was improved, which was conducive to increasing the compactness of the internal structure.
5. The research results might lay a foundation for further revealing the improving mechanism of SAP internal curing concrete in actual working environments, and also provide a theoretical basis for enhancing the durability of pavement concrete. The appropriate dosage of SAP was 0.150% with the *W*/*C* of 0.4.

In order to predict the service life of SAP-concrete under the coupling effect of salt freeze–thaw, a life prediction model can be established with multiple parameters. What is more, to better fit the actual engineering conditions, the characteristics of the soil, such as saline soil areas, can be taken into account. Further research of the performance degradation and influencing mechanism of SAP internal curing cement-based materials under multiple coupling environments such as sulfate dry–wet and salt freeze–thaw can be carried out.

## Figures and Tables

**Table 1:** Main technical indicators of SAP.

Exterior		White Irregular Powder
Analytical (chemical) purity (%)		92
Density (g/cm^3^)		0.7–0.75
Water absorption (deionized water; g/g)		450–550
Saturated absorption time (deionized water; s)		<28
pH (1% moisture dispersion)		5.5–6.8
Water retention rate (%)		>96
Liquid absorption (g/g)	100–120 mesh	24.8
	150–200 mesh	22.6

**Table 2:** Physical and mechanical properties of cement.

Value Type	Testing Indexes					
	Fineness /(m^2^/kg)	Loss of Ignition/%	Initial Set Time /Min	Final Set Time /Min	Flexural Strength on Day 3/MPa	Compressive Strength on Day 3/MPa
Standard value	≥300	≤5.0	≥45	≤600	≥3.5	≥17.0
Measured value	358	3.31	172	234	5.5	27.2

**Table 3:** Mix proportion of mortar.

Mortar Type	SAP	IC Water/(Kg/m^3^) (By Mass of Cement)	SAP Dosage/% (By Mass of Cement)	Cement:Sand: Water Reducer
Control	-	-	-	100:210:0.5
SAP-1-0.10%	SAP-100	0.025	0.10	
SAP-1-0.15%		0.037	0.15	
SAP-1-0.20%		0.050	0.20	
WRA-1-0.15%		0.037	0.15	
SAP-2-0.10%	SAP-200	0.023	0.10	
SAP-2-0.15%		0.034	0.15	
SAP-2-0.20%		0.045	0.20	
WRA-2-0.15%		0.034	0.15	

**Table 4:** Performance test results of mortar.

Mortar Type	Flexural Strength/MPa	Compressive Strength/MPa	Shrinkage Rate on Day 3/%	Shrinkage Interval Length from Day 14 to Day 28/%
Control	8.4	42.0	0.0383	0.0268
SAP-1-0.10%	7.8	38.1	0.0224	0.0079
SAP-1-0.15%	8.3	40.0	0.0208	0.0046
SAP-1-0.20%	7.5	35.0	0.0228	0.0065
WRA-1-0.15%	8.0	35.9	0.0202	0.0043
SAP-2-0.10%	7.4	38.7	0.0212	0.0140
SAP-2-0.15%	7.7	41.1	0.0226	0.0178
SAP-2-0.20%	7.0	35.4	0.0237	0.0187
WRA-2-0.15%	7.8	37.0	0.0189	0.017

**Table 5:** Target degree of mortar.

Mortar type	Control	SAP-1 −0.10%	SAP-1 −0.15%	SAP-1 −0.20%	WRA-1 −0.15%	SAP-2 −0.10%	SAP-2 −0.15%	SAP-2 −0.20%	WRA-2 −0.15%
Target degree	0.704	0.725	0.890	0.697	0.874	0.705	0.723	0.624	0.756

**Table 6:** Mix proportion of internal curing pavement concrete after optimization.

Concrete Type	*W_IC_* /(kg/m^3^)	SAP Dosage /kg	Compositions of Pavement Concrete/(kg/m^3^)					
			Cement	Water	Sand	5–10 mm Coarse	10–20 mm Coarse	Water Reducer
Control	-	-	360	144	756	349	814	4.32
SAP-0.125%	11.16	0.45						
SAP-0.150%	13.39	0.54						
SAP-0.175%	15.62	0.63						

**Table 7:** Total surface area and average pore diameter before salt freeze–thaw test.

Parameter		Specimen		
	Control	SAP-0.125%	SAP-0.150%	SAP-0.175%
Total surface area (m^2^·g^−1^)	2.5047	4.0843	5.5079	5.9894
Average pore size (nm)	46.07	44.07	37.12	36.18

**Table 8:** Statistical summary diagram of pores.

Concrete Type	Cycles/Time	Count/Pcs	Total Area/ Pixel ²	Average Size/ Pixel ²	%Area/%
Control	0	267	511	1.914	0.079
	25	263	932	3.544	0.145
	50	1218	6794	5.578	1.056
	75	3152	17,434	5.531	2.715
SAP-0.150%	0	103	632	6.136	0.097
	25	292	721	2.649	0.112
	50	618	4163	6.736	0.646
	75	716	8025	11.208	1.250

**Table 9:** Statistical summary diagram of pores with different dosage in 75 cycles.

Specimen	Control	SAP-0.125%	SAP-0.150%	SAP-0.175%
Count/pcs	3152	2718	716	5822
Total Area/pixel ^2^	17,434	17,848	8025	20,472
Average Size/pixel ^2^	5.531	6.567	11.208	3.516
%Area/%	2.715	1.560	1.250	1.788

## Data Availability

Not applicable.
